# Prevalence of lumbosacral transitional vertebrae and association with hip dysplasia in Rhodesian Ridgebacks in Germany

**DOI:** 10.17221/30/2025-VETMED

**Published:** 2025-10-29

**Authors:** Svenja Kristiane Slunsky, Pavel Slunsky, Emanuel Fort, Leo Brunnberg

**Affiliations:** ^1^Neurological Department, Small Animal Hospital, AniCura Tierklinik Haar, Haar, Bavaria, Germany; ^2^Department of Surgery and Orthopaedics, Faculty of Veterinary Medicine, University of Veterinary Sciences Brno, Brno, Czech Republic; ^3^Joint Research Unit for Epidemiology and Translational Surveillance, Claude Bernard University Lyon, Villeurbanne, France, Claude Bernard University Lyon, Villeurbanne, France; ^4^Department of Veterinary Medicine, Small Animal Clinic, Freie Universität Berlin, Berlin, Germany

**Keywords:** breed-specific conditions, canine orthopaedics, imaging diagnostics, musculoskeletal research, spinal malformations, vertebral classification

## Abstract

Congenital anomalies affecting the spinal column are frequently observed in certain dog breeds. One such condition occurs at the junction between the lower back and the *sacrum*, where vertebrae exhibit mixed structural characteristics. This retrospective study analysed imaging records of Rhodesian Ridgebacks collected over 28 years, selecting only those that met strict positioning standards for evaluation. A total of 2 012 cases were analysed using established classification methods, identifying three distinct structural variations: a typical junction, a bilaterally altered morphology, and an asymmetrical form affecting one side. The prevalence of these variations was 7.4%, with symmetrical alterations found in 5% of cases and asymmetrical alterations in 2.4%. Statistical analysis did not reveal a significant association between these anatomical variations and canine hip dysplasia (CHD) (*P* = 0.170 0). These findings support previous research indicating that there is no direct link between these structural variations and joint disorders in this breed. The study represents the most extensive investigation of its kind in this canine population, highlighting that such vertebral variations are frequently observed in routine radiographic screenings, but despite decades of research, consistent clinical correlations remain elusive – underscoring the need for further systematic investigation.

Lumbosacral transitional vertebrae (LTV) are common congenital anomalies in various dog breeds (Morgan 1968; Morgan et al. 1993; Morgan 1999b; Fluckiger et al. 2006). They occur at the boundary between the lumbar spine and *sacrum* and exhibit characteristics of both lumbar and sacral vertebrae. Additionally, there may be an intervertebral space between the first and second sacral vertebrae (Morgan 1999a; Morgan 1999b). Lumbosacral transitional vertebrae (LTV) can present with asymmetrical vertebral processes and may alter the morphology of adjacent spinal segments, resulting in varying degrees of lumbarisation or sacralisation (Damur-Djuric et al. 2006). A heritability of LTV is suspected (Larsen 1977; Morgan and Stephens 1985; Winkler 1985; Ziegler 1989). Asymmetrical LTV may lead to deviations in the spinal axis, resulting in uneven load distribution on the hip and sacroiliac joints as well as the pelvic limbs (Schawalder et al. 1996; Dietschi et al. 2000; Fluckiger et al. 2017). The connection between LTV and hip dysplasia has not yet been clearly established (Larsen 1977; Winkler 1985; Owens 1989; Ziegler 1989; Tellhelm and Brass 1994).

This study aims to investigate the prevalence of LTV in Rhodesian Ridgebacks and their potential predisposition to canine hip dysplasia (CHD, canine hip dysplasia).

## MATERIAL AND METHODS

For this retrospective study, analogue radiographs of Rhodesian Ridgebacks taken between December 18, 1987 and July 17, 2015 were evaluated. These images were taken at Small Animal Clinic, Department of Veterinary Medicine, Free University of Berlin (Berlin, Germany) or elsewhere and sent to the clinic for evaluation. The pelvic radiographs were obtained in the dorsal recumbency position with the hind limbs extended, in accordance with the requirements for canine hip dysplasia (CHD) diagnostics (FCI Position 1).

Only radiographs of the pelvis in CHD Position I, where the lumbosacral transition area was fully visible and the animal was correctly positioned, were evaluated. Radiographs of poor quality due to incorrect positioning, technical errors, overlaid by dense faecal matter, or the dense penis bone, or those incorrectly labelled, were excluded from the study.

The identity of the animals was determined from the inscription on the radiographs and the CHD assessment form, which included breed registry information, animal name, sex, breed book number, birth date, chip number and/or tattoo number, the owner’s name and address, and the date of the radiographic examination. The age of the animals at the time of the radiographs was calculated based on their birth and radiograph dates.

The radiographs were assessed by two of the authors (SS, LB). LB is a certified radiologist and the breed’s responsible CHD evaluator; SS is a veterinary medicine doctor. The diagnosis of lumbosacral transitional vertebra (LTV) was made by consensus. The hip joints were evaluated according to the criteria of the Federation Cynologique Internationale (FCI) by the breed’s responsible CHD evaluator (LB).

The pelvic radiographs were examined for changes in the lumbosacral transition area in the form of transitional vertebrae. If morphological changes were observed, they were documented and classified according to Julier-Franz (2006).

To analyse the morphology of the lumbosacral junction, the shape of the last lumbar vertebra (L7), the *sacrum,* and the intervertebral space between L7 and S1 were assessed as follows:

The transverse processes of L7 were evaluated based on their length, orientation, and contact with the *sacrum*: Classification was performed in three categories:

- Length (Grade 0 indicated normally developed transverse processes, Grade 1 indicated significantly shortened processes, Grade 2 indicated rudimentary processes, and Grade 3 indicated fusion with the *sacrum* or complete absence);
- Orientation (Grade 0 indicated craniolateral orientation, Grade 1 indicated lateral orientation, and Grade 2 indicated no discernible orientation);
- Contact with the *sacrum* (Grade 0 indicated no contact, Grade 1 indicated slight caudal elongation, Grade 2 indicated recognisable but still distinguishable contact, and Grade 3 indicated complete fusion where the transverse process could no longer be identified as a separate structure).

The morphology of the *sacrum* was assessed by counting the number of sacral vertebrae and measuring the interspinal space. Three fused sacral vertebrae were classified as Grade 0, two as Grade 1, and four as Grade 2. The interspinal space was considered normal (Grade 0) or enlarged between the first and second spinous processes (Grade 1).

The intervertebral space between L7 and S1 was categorised as follows: Grade 0 indicated a normal width of the intervertebral gap, Grade 1 indicated lateral contact between L7 and S1 with an open central intervertebral space, Grade 2 indicated the intervertebral space appearing as a radio-opaque line, and Grade 3 indicated complete fusion of L7 with the *sacrum*.

Based on these criteria, three forms of the lumbosacral transition region were identified: 0) anatomically “normal” lumbosacral junction, 1) symmetrical lumbosacral transitional vertebra (symLTV), with Grade 1a (isolated spinous process of S1) and Grade 1b (bilaterally symmetrical morphological deviations of L7 or S1, where the transitional vertebra is completely separated from the *sacrum*), and 2) asymmetrical lumbosacral transitional vertebra (asymLTV), either right- or left-sided, with an abnormal right-sided transverse process without (2a) or with contact to the *ilium* (2b) or an abnormal left-sided transverse process without (2c) or with contact to the *ilium* (2d) respectively. For this study, the classification scheme proposed by Julier-Franz (2006), which allows for more detailed differentiation, was used.

The results were statistically analysed for correlation with hip dysplasia grades.

To improve comparability with previous studies, the findings of this study were contextualised according to the classification system proposed by Fluckiger et al. (2017), which remains one of the most widely used schemes for LTV morphology. While direct equivalence is not possible due to differing definitions, general alignment can be observed. The absence of transitional morphology in our dataset corresponds to non-LTV cases in the Flueckiger system.

Grade 1a (isolated spinous process of S1) was not included in the Flueckiger classification, as such cases were not considered transitional vertebrae. Grade 1b (symmetrical transverse processes without sacroiliac contact) may correspond approximately to combinations such as Type 2/2 in the Flueckiger scheme. Asymmetrical LTV (asymLTV), characterised by differing morphology between left and right transverse processes, aligns with Flueckiger combinations such as 1/2, 2/3, or 1/3, which indicate unilateral or asymmetrical sacroiliac articulation.

While these comparisons provide a general framework for comparison, key differences remain in criteria – notably concerning spinous process variants and sacral segmentation – and preclude one-to-one equivalence between systems.

### Statistical analysis

Both descriptive and stratified statistical analyses were performed. Qualitative variables were compared using the chi-square test. The homogeneity of variance was first tested using Bartlett’s test. Percentage values were rounded to one decimal place.

## RESULTS

### Descriptive findings

A total of 2 037 canine hip dysplasia (CHD) radiographs from Rhodesian Ridgebacks were evaluated, including 839 males (41.2%) and 1 198 females (58.8%). The mean age at the time of radiographic examination was 20.0 months (range 12–136), and the median age was 17 months. The mean and median age at the time of radiographic examination were recorded and are presented descriptively (mean, median, range). No statistical comparison of age between groups was performed, as LTV is a congenital anatomical variation not expected to vary with age.

A total of 2 012 dogs (826 males, 41.1%; 1 183 females, 58.9%) met the radiological criteria for assessing the lumbosacral transitional region. A physiological lumbosacral transitional region was present in 1 863 dogs (92.6%) (767 males, 41.1%; 1 096 females, 58.9%).

Lumbosacral transitional vertebrae (LTV) were detected in 149 dogs (7.4%) (59 males, 39.6%; 90 females, 60.4%). No significant correlation was found between sex and LTV (*P* = 0.707 3).

A symmetrical LTV (Grade 1) was present in 101 dogs (5.0%) (41 males, 40.6%; 60 females, 59.4%). Among them, 43 dogs (2.1%) exhibited an isolated spinous process of S1 (Grade 1a, Flueckiger Type I), and 58 dogs (2.9%) showed bilateral symmetrical morphological changes of L7 or S1 (Grade 1b, Flueckiger Type II). An asymmetrical LTV (Grade 2) was detected in 48 dogs (2.4%) (18 males, 37.5%; 30 females, 62.5%). In 8 dogs (0.4%), no contact with the *ilium* was visible (Grade 2a). In 40 dogs (2.0%), the transitional process made radiographic contact with the *ilium* (Grade 2b, Flueckiger Type III).

An asymmetrical LTV was identified in a total of 48 dogs (2.4%) (18 males, 37.5%; 30 females, 62.5%). In 23 dogs (1.1%), the transverse process was abnormally developed on the left side (10 males, 43.5%; 13 females, 56.5%), while in 25 dogs (1.2%), it was abnormal on the right side (8 males, 32%; 17 females, 68%). In 8 dogs (0.4%), an asymmetrical LTV was present without detectable contact with the *ilium* (4 males, 50%; 4 females, 50%). In 40 dogs (2%), an asymmetrical transitional vertebra was in contact with the *ilium* (14 males, 35%; 26 females, 65%).

No significant correlation was found between sex and symmetrical LTV (*P = *0.923 2) or asymmetrical LTV (*P* = 0.635 0) (Table 1).

**Table 1:** Prevalence of lumbosacral transitional vertebrae (LTV) in Rhodesian Ridgebacks

Group	Male [*n* (%)]	Female [*n* (%)]	Total [*n* (%)]
Normal	767 (41.2)	1 096 (58.9)	1 863 (92.6)
LTV	59 (39.6)	90 (60.4)	149 (7.4)
SymLTV	41 (40.6)	60 (59.4)	101 (5)
Type 1a (isolated spinous process of S1)	17 (39.5)	26 (60.5)	43 (2.1)
Type 1b (expanded symLTV)	24 (41.4)	34 (58.6)	58 (2.9)
AsymLTV	18 (37.5)	30 (62.5)	48 (2.4)
Type 2a (without contact to *ilium*)	4 (50)	4 (50)	8 (0.4)
Type 2b (contact with *ilium*)	14 (35)	26 (65)	40 (2)
			
Total [*n* (%)]	826 (41.1)	1 186 (58.9)	2 012 (100)

Percentages refer to row totals within each category; Type 1a = isolated spinous process of S1 (not included in the Flueckiger classification); Type 1b = bilaterally enlarged transverse processes without ilial contact (corresponds approximately to Flueckiger Type II); Type 2a = asymmetrical LTV without contact to the *ilium* (Flueckiger combinations such as 1/2 or 2/3); Type 2b = asymmetrical LTV with contact to the *ilium* (Flueckiger combination such as 1/3)

asym = asymmetrical; sym = symmetrical

Radiographs from 2 012 Rhodesian Ridgebacks met the necessary criteria for evaluating CHD status (826 males, 41%; 1 187 females, 59%). A total of 1 688 dogs (83.8%) were classified as CHD-free (Grade A) (683 males, 40.5%; 1 005 females, 59.5%). Canine hip dysplasia (CHD) was diagnosed in 325 dogs (16.2%) (143 males, 44%; 182 females, 56%) (Table 2).

**Table 2:** Canine hip dysplasia (CHD) grades in Rhodesian Ridgebacks

CHD grade	Male [*n* (%)]	Female [*n* (%)]	Total [*n* (%)]
Normal (CHD Grade A)	683 (40.5)	1 005 (59.5)	1 688 (83.8)
CHD (Grades B–E)	143 (44)	182 (59.5)	325 (16.2)
CHD Grade B	95 (43.4)	124 (56.6)	219 (10.9)
CHD Grade C	35 (43.7)	45 (56.3)	80 (4)
CHD Grade D	8 (50)	8 (50)	16 (0.8)
CHD Grade E	5 (50)	5 (50)	10 (0.5)
			
Total [*n* (%)]	826 (41.1)	1 186 (58.9)	2 012 (100)

Percentages refer to row totals within each category; Grades A–E were classified according to the official dysplasia scoring scheme; CHD Grade A = no signs of dysplasia; Grades B–E indicate increasing severity of dysplasia (from mild to severe)

CHD = canine hip dysplasia

### Analytical findings

No significant correlation was found between LTV and CHD (*P* = 0.170) (Table 3).

**Table 3:** Distribution of lumbosacral transitional vertebrae (LTV) and canine hip dysplasia (CHD) in Rhodesian Ridgebacks

Group	CHD Grade A [*n* (%)]	CHD Grade B–E [*n* (%)]	Total [*n* (%)]
No LTV	1 568 (84.2)	295 (15.8)	1 863 (92.6)
LTV	119 (79.9)	30 (20.1)	149 (7.4)

Percentages refer to row totals within each LTV group; CHD Grade A indicates no signs of dysplasia; CHD Grades B–E were classified as affected (increasing severity from mild to severe)

CHD = canine hip dysplasia; no LTV = no lumbosacral transitional vertebrae observed

Additionally, no significant correlation was observed between symmetrical LTV and CHD (*P* = 0.119 36) or between the different forms of symmetrical LTV and CHD (*P = *0.106) (Table 4).

**Table 4:** Symmetrical lumbosacral transitional vertebrae (symLTV) and canine hip dysplasia (CHD) in Rhodesian Ridgebacks

Group	CHD Grade A [*n* (%)]	CHD Grades B–E [*n* (%)]	Total [*n* (%)]
No symLTV	1 607 (84.1)	304 (15.9)	1 911 (95)
SymLTV	80 (79.2)	21 (20.8)	101 (5)
Type 1a (isolated spinous process of S1)	31 (72.1)	12 (27.9)	43 (2.1)
Type 1b (expanded symLTV)	49 (84.5)	9 (15.5)	58 (2.9)
			
Total [*n* (%)]	1 687 (83.9)	325 (16.1)	2 012 (100)

Percentages refer to the column total of each CHD category; CHD Grades B–E were classified as affected

CHD = canine hip dysplasia; symLTV = symmetrical lumbosacral transitional vertebrae; Type 1a = isolated spinous process of S1 (not included in the Flueckiger classification); Type 1b = enlarged transverse processes without contact to the *ilium* (corresponds approximately to Flueckiger Type 2/2)

Similarly, no significant correlation was found between asymmetrical LTV and CHD (*P* = 0.620). Moreover, no significant correlation was detected between asymmetrical LTV with contact to the *ilium* and CHD (*P* = 0.254) (Table 5).

**Table 5:** Asymmetrical lumbosacral transitional vertebrae (asymLTV) and canine hip dysplasia (CHD) in Rhodesian Ridgebacks

Group	CHD Grade A [*n* (%)]	CHD Grades B–E [*n* (%)]	Total [*n* (%)]
No asymLTV	1 648 (83.9)	316 (16.1)	1 964 (97.6)
AsymLTV	39 (81.3)	9 (18.7)	48 (2.4)
Type 2a (without contact to *ilium*)	8 (100)	0 (0)	8 (0,4)
Type 2b (contact with *ilium*)	31 (77.5)	9 (22.5)	40 (2)
			
Total [*n* (%)]	1 687 (83.9)	325 (16.1)	2 012 (100)

Percentages refer to row totals within each asymLTV group; CHD Grades B–E were classified as affected

asymLTV = asymmetrical lumbosacral transitional vertebrae; CHD = canine hip dysplasia; Type 2a = asymmetrical LTV without contact to the *ilium*; Type 2b = asymmetrical LTV with contact to the *ilium* (corresponds approximately to Flueckiger combination 1/3)

For clarity, Table 6 summarises the *P*-values for the associations between the various LTV subtypes and CHD, emphasising the lack of statistically significant correlations in this cohort.

**Table 6:** Statistical association between lumbosacral transitional vertebrae (LTV) and canine hip dysplasia (CHD) in Rhodesian Ridgebacks (*P*-values)

Group	CHD
LTV	*P* = 0.170
SymLTV	*P* = 0.119
Subtypes 1a and 1b	*P* = 0.106
AsymLTV	*P* = 0.620
Subtypes 2a and 2b	*P* = 0.254

Statistical significance was assessed using chi-square tests (based on *P*-values)

asymLTV = asymmetrical lumbosacral transitional vertebrae (subtypes 2a and 2b: without or with contact to the *ilium*); CHD = canine hip dysplasia; symLTV = symmetrical lumbosacral transitional vertebrae (subtypes 1a and 1b: isolated spinous process of S1, enlarged transverse processes without iliac contact)

## DISCUSSION

### Prevalence of LTV in Rhodesian Ridgebacks in the literature

The reported prevalence of lumbosacral transitional vertebrae (LTV) varies widely in the literature, ranging from 0% to 46.2%, depending on the study and breed. However, standardised classification methods and a consensus on nomenclature are still lacking (Larsen 1977; Winkler 1985; Morgan and Stephens 1985; Ziegler 1989; Herling 1996; Breit et al. 2003; Damur-Djuric et al. 2006; Julier-Franz 2006; Ledecky et al. 2007; Lappalainen et al. 2012; Fialova et al. 2014; Moeser and Wade 2017; Brocal et al. 2018; Kuricova et al. 2018; Gong et al. 2020; Gluding et al. 2021; Berg et al. 2024; Kim et al. 2025).

In the present study, LTV was found in 7.4% (149/2 012) of Rhodesian Ridgebacks. An isolated *processus spinosus* S1 was detected in 2.1% (43/2 012), while a distinct symmetrical transitional vertebra was observed in 2.9% (58/2 012). In total, 5% (101/2 012) had a symmetrical LTV, and 2.4% (48/2 012) had an asymmetrical LTV.

Data on the prevalence of LTV in Rhodesian Ridgebacks are available from Larsen (1977), Winkler (1985), Ledecky et al. (2007), Fialova et al. (2014), and Berg et al. (2024). However, these studies used different classification and typification models.

Larsen (1977) found LTV in 7.9% (15/191) of Rho-desian Ridgebacks, indicating that this breed, along with German Shepherds, Brittany Spaniels, and Doberman Pinschers, had a significantly higher prevalence of LTV than other breeds. Larsen (1977) did not specify criteria for distinguishing between lumbarisation and sacralisation, but suggested that sacralisation of the seventh lumbar vertebra was the most common type although the total number of pre-sacral vertebrae remained unknown. This pattern was also observed in other breeds, where only CHD radiographs in FCI Position I and/or II were available for assessment. The lack of consistent imaging of the lumbosacral transition area led Larsen (1977) to suspect a higher true prevalence, a hypothesis later supported by Winkler (1985). Winkler (1985) also evaluated CHD radiographs and found LTV in 16.7% (4/24) of Rhodesian Ridgebacks. Like Larsen (1977), he used the terms lumbarisation and sacralisation, further distinguishing sacralisation into unilateral and bilateral forms. Ledecky et al. (2007) reported an LTV prevalence of 2.12% (1/47) among the examined Rhodesian Ridgebacks. Their study grouped LTV into symmetrical and asymmetrical types but did not specify which types were found in each breed. It is unclear whether an isolated *processus spinosus* S1 was considered. Fialova et al. (2014) analysed pelvic radiographs in ventrodorsal and laterolateral projections, finding an LTV prevalence of 15.7% (14/89) in Rhodesian Ridgebacks. Following Damur-Djuric et al. (2006), they assessed the intervertebral space between the first two sacral vertebrae using the classification by Fluckiger et al. (2009): Type 0 (normal lumbosacral transition), Type 1 (isolated *processus spinosus* S1), Type 2 (LTV fully separated from the *sacrum* with symmetrical transverse processes), and Type 3 (asymmetrical transverse processes). Their study identified Type I LTV in 1.1% (1/89), Type II in 10.1% (9/89), and Type III in 4.5% (4/89). Berg et al. (2024) also classified LTV using Fluckiger et al. (2009) and found LTV in 43.1% (216/501) of Rhodesian Ridgebacks, with 14.0% (70/501) having Type I, 23.2% (118/501) having Type II, and 6.0% (30/501) having Type III.

### Sex predisposition

In the present study, no sex predisposition for LTV was identified in Rhodesian Ridgebacks (*n* = 2 012, *P* = 0.707). This finding is consistent with previous literature on Rhodesian Ridgebacks (Larsen 1977, *n* = 191; Winkler 1985, *n* = 24; Ledecky et al. 2007, *n* = 47; Fialova et al. 2014, *n* = 89).

### Lumbosacral transitional vertebrae (LTV) and canine hip dysplasia (CHD)

Lumbosacral transitional vertebrae are frequently observed as incidental findings during CHD screening, which is mandatory in Germany for several dog breeds as part of breeding approval. These radiographs have revealed vertebral abnormalities at the lumbosacral transition in multiple breeds, often labelled as incidental findings. Their clinical significance remains uncertain.

The radiographs in this study were obtained during CHD screenings according to FCI criteria, with CHD grades ranging from A (CHD-free) to E (severe CHD). Canine hip dysplasia (CHD; grade > A) was diagnosed in 16.2% (325/2 012) of Rhodesian Ridgebacks. Statistically, no significant association between LTV and CHD was detected, nor between specific LTV subtypes (isolated *processus spinosus* S1, distinct symmetrical LTV, or asymmetrical LTV) and CHD.

These findings align with previous studies by Larsen (1977), Winkler (1985), Winkler and Loeffler (1986), Tellhelm and Brass (1994), Morgan (1999a), Citi et al. (2005), Julier-Franz (2006), Ledecky et al. (2007), Wigger et al. (2009), and Kuricova et al. (2018), all of which found no correlation between LTV and CHD in 57 dog breeds.

However, other studies (Morgan and Stephens 1985; Keller and Corley 1989; Owens 1989; Ziegler 1989; Schawalder et al. 1996; Dietschi et al. 2000; Komsta et al. 2015; Fluckiger et al. 2017) demonstrated significant correlations between LTV and CHD in 85 breeds. Recently Berg et al. (2024) examined 14 breeds, including Rhodesian Ridgebacks, and found a statistically significant association between LTV Type 2 and Type 3 and CHD severity, but specific results for Rhodesian Ridgebacks were not detailed (Table 7).

**Table 7:** Selected studies reporting a significant association between LTV and CHD in dogs

Author	Significant correlation
Morgan and Stephens (1985)	asymmetrical pelvic connection to *sacrum* correlated with CHD
Keller and Corley (1989); Schawalder et al. (1996); Dietschi et al. (2000)	asymmetrical LTV correlated with ipsilateral CHD
Ziegler (1989)	symmetrical LTV with bilateral CHD and coxarthrosis; asymmetrical LTV ipsilateral with severe coxarthrosis
Owens (1989)	asymmetrical LTV in contact with the *ilium* associated with CHD and contralateral coxarthrosis
Komsta et al. (2015)	LTV associated with a higher frequency of severe CHD
Fluckiger et al. (2017)	type 2/3 asymmetrical LTV significantly associated with femoral head subluxation
Lang and Jaggy (1989); Ziegler (1989); Fluckiger et al. (2017)	LTV associated with spinal axis deviation and/or pelvic rotation; asymmetrical LTV may predispose to CHD if pelvic rotation is present and contact with the *ilium* occurs; without contact, hip joints remain normally aligned
Berg et al. (2024)	LTV types 2 and 3 with both mild and severe CHD

This table summarises studies reporting significant associations between lumbosacral transitional vertebrae (LTV) and canine hip dysplasia (CHD). However, not all studies have confirmed such correlations; conflicting results exist in the literature

### Overvaluation of the lumbosacral transitional vertebrae (LTV) topic

The topic of lumbosacral transitional vertebrae (LTV) remains a recurrent issue in veterinary medical research. Despite numerous studies addressing the prevalence, classification, and potential clinical implications of LTV, the question of whether these changes actually have clinical relevance remains unresolved. In the present study, despite the largest sample size for the Rhodesian Ridgeback breed (*n* = 2 012), no significant correlation between LTV and hip dysplasia (CHD) was found. These results match with previous work, such as that by Larsen (1977), Winkler (1985), and Fialova et al. (2014), who also found no clear connection between LTV and hip dysplasia.

A key point in the debate surrounding LTV is the uncertainty about the clinical significance of these changes. Many of the identified LTVs appear as “incidental findings” during radiological examinations, such as hip dysplasia screening X-rays (Larsen 1977; Winkler 1985; Fialova et al. 2014). These results raise the question of whether the diagnostic effort for LTV is justified when the clinical consequences remain so unclear. Numerous studies have shown that LTV often has no or only minimal impact on the quality of life of the animals (Moeser and Wade 2017; Brocal et al. 2018). Berg et al. (2025a) reported a high prevalence of LTV in their cohort, but they could not establish a statistically significant correlation with any clinical condition. Therefore, the widespread diagnosis of LTV might be more of a remnant from breeding and screening traditions rather than a clinically relevant entity.

### Classification systems and diagnostic uncertainties

Another central issue when interpreting the literature is the lack of a unified classification system for LTV. In the present study, a classification proposed by Fluckiger et al. (2009) was used, which distinguishes between various types of LTV based on the symmetry and structural features of the transitional vertebrae. Unfortunately, this classification has not been consistently applied across the literature. Larsen (1977) used an imprecise terminology that differentiates between “lumbarisation” and “sacralisation” without clear criteria, which can lead to misunderstandings in diagnosis and interpretation. Similarly, Winkler (1985) reports a distinction between unilateral and bilateral sacralisation, which, however, is not always consistent in other studies. This makes direct comparisons between studies difficult and hampers the ability to draw generalisable conclusions.

The classifications based on radiographs are also inherently limited. As emphasised by Fialova et al. (2014), lumbosacral transitional vertebrae may be assessed differently in various radiographic projections. In particular, in the ventrodorsal projection, even the smallest anatomical changes, such as the contact points between the vertebrae, can easily be overlooked. A more precise diagnosis can only be achieved through computed tomography (CT), which allows for a more accurate assessment. However, this is associated with considerable diagnostic effort, as prolonged anaesthesia is required, raising ethical concerns, especially since the clinical relevance of these changes remains questionable (Ziegler 1989; Morgan et al. 1999b). Additionally, the question remains whether this effort is justified when there has been no clear evidence of clinical significance for LTV for decades.

### Genetic aspects and regional differences

Another aspect to consider in the discussion is the possible genetic predisposition for LTV, particularly evident in certain populations. Berg et al. (2024) found a high prevalence of LTV in their Norwegian study, suggesting that genetic factors may play a role in the development of LTV. This regional variation could indicate that certain geographic areas or breeding populations may exhibit a higher occurrence of LTV. The lower prevalence of LTV observed in some populations – such as the German Rhodesian Ridgebacks evaluated in this study – may be partly explained by national breeding regulations.

In Germany, dogs with moderate or severe CHD (grades C to E) are excluded from breeding, and dogs with lumbosacral transitional vertebrae (LTV) may only be bred under strict conditions – specifically, with unaffected partners and after offspring evaluation (RRCD 2023).

Recent studies confirm a genetic influence on the occurrence of lumbosacral transitional vertebrae (LTV) in dogs. For example, Berg et al. (2025a) and Berg et al. (2025b) estimated heritability values for LTV ranging from low to moderate (0.056–0.314) across nine breeds. These findings indicate that although LTV is only moderately heritable, genetic selection could potentially reduce its prevalence in affected populations. In contrast, the heritability estimates for CHD were moderate to high (0.254–0.580). Importantly, no general genetic correlation was found between LTV and CHD, suggesting that these two conditions may segregate independently within breeds.

However, it remains unclear whether these anatomical changes are truly associated with clinical symptoms, such as pain or restricted movement. The high prevalence in some populations, such as Rhodesian Ridgebacks in the Norwegian study, may point to a breed-related accumulation of LTV without necessarily being linked to any actual disease burden.

### Study limitation

This study was based on retrospective radiographic data obtained through official CHD screening procedures. No clinical follow-up or information regarding the dog’s use (e.g., sport or working activity) was available. It is also possible that dogs with known symptoms or radiographic abnormalities were not submitted for screening, which introduces a potential selection bias. Additionally, classification was limited to standard radiographs; lateral views and advanced imaging techniques such as CT were not employed.

In conclusion, it can be stated that the topic of LTV still garners some attention in veterinary medical research, but there is no clear and consistent evidence of clinical relevance. The high prevalence of LTV in certain breeds and populations, such as the Rhodesian Ridgebacks in the present study, rather seems to be an indicator of genetic predisposition. However, it remains questionable whether the diagnostic effort and research on LTV are justified in cases with unclear clinical impact, and no significant correlation with other conditions, such as canine hip dysplasia, has been established. A unified classification system and more accurate diagnostic methods, such as computed tomography, could contribute to better assessment, but the question remains whether the associated effort for a condition whose significance remains unproven is truly justified.
